# Editorial: Extracellular vesicle-derived non-coding RNAs (EV-ncRNAs) and their multifaceted roles in cancer biology

**DOI:** 10.3389/fonc.2023.1185363

**Published:** 2023-06-02

**Authors:** Nahid Neamati, Atiyeh Al-e-Ahmad, Farshid Yeganeh, S. Mohsen Asghari, Hadi Parsian

**Affiliations:** ^1^ Systems Biomedicine Unit, Pasteur Institute of Iran, Tehran, Iran; ^2^ Department of Clinical Biochemistry, Faculty of Medicine, Babol University of Medical Sciences, Babol, Iran; ^3^ Student Research Committee, Babol University of Medical Sciences, Babol, Iran; ^4^ Department of Medical Immunology, School of Medicine, Shahid Beheshti University of Medical Sciences, Tehran, Iran; ^5^ Institute of Biochemistry and Biophysics (IBB), University of Tehran, Tehran, Iran; ^6^ Cellular and Molecular Biology Research Center, Health Research Institute, Babol University of Medical Sciences, Babol, Iran

**Keywords:** extracellular vesicle (EV), non-coding RNAs (ncRNAs), extracellular vesicle-derived non-coding RNAs (EV-ncRNAs), cancer, exosome

Cell-cell interactions and intracellular communication are fundamental circumstances in a wide range of biological and pathological events. As a proven fact, in biological systems, the communication between cells and their surrounding microenvironment requires the presence of messenger molecules, cytokines, and neurotransmitters, as well as intercellular exchanges through extracellular messenger vesicles with an endosomal origin.

Extracellular vesicles (EVs), especially exosomes, can act as vehicles for intracellular content such as proteins, enzymes and even nucleic acids, i.e. DNA and RNA wherein they can be protected from nucleases, proteases and other degrading substances. Exosomes can be released into body fluids and spared (1, 2). Thus, they are potent carriers of intracellular cargo to nearby and distant targets as well. Structurally, EVs are endosomally originate with bilayer lipid membranes containing unique superficial proteins and CD-markers (3). According to size and origin, EVs are subdivided into microvesicles, exosomes and apoptotic bodies. Apoptotic bodies are variable in size, ranged between 50nm up to 5µm in diameter and originate from dying cells. Microvesicles have typically smaller size (100nm up to 1µm in diameter). Like exosomes, microvesicles are involved in cellular communications. The smallest kind of EVs are exosomes, those have a diameter size between 30–150nm and originated from endosome (4). A growing body of evidence implies EVs are potent nanoparticles for research in the pathogenesis of the disease or discovering new diagnostic, in addition to prognostic or therapeutic target candidate biomarkers. Studying of EVs is of important since they can be released into body fluids and are detectable by relatively simple laboratory techniques (5). As EVs have intracellular origin, valuable information can be achieved through their studying. In addition, the study on EVs’ cargoes including various lipids, proteins, metabolites and nucleic acids is a worthy aspect of clinical research (6, 7).

The nucleic acid content of EVs including double-stranded DNAs (dsDNA), messenger RNAs (mRNA), long and short non-coding RNAs, (i.e. long non-coding RNAs (lncRNA), microRNAs (miRNA), small interfering RNA (siRNA), circular RNAs (circRNAs), etc.) are suggested as functional messengers for initiation and progression of cancer or even drug resistance. Cancer initiation, survival and progression are strongly influenced by the communications between cells and the tumor microenvironment. As mentioned by Zhang et al. lots of non-coding RNAs can be packaged and transferred within EVs and influence the intra/extracellular signaling pathway. These findings highlight the functional role of EV-ncRNAs in cancer biology.

This Research Topic focuses on *extracellular vesicle-derived non-coding RNAs (EV-ncRNAs) and their multifaceted roles in cancer biology*. Exsosomal lncRNA ANCR was introduced by Hu et al. as a potential therapeutic target for osteosarcoma because of its functional act in resistance to doxycycline. Sadovska et al. in a comprehensive study reported valuable results about the diagnostic and prognostic value of EV-ncRNAs in breast cancer patients (BCa) undergoing neoadjuvant chemotherapy. In their study, serum EVs were extracted at the initial, 7^th^ day as well as 6^th^ and 12^th^ months after surgery from patients with breast cancer versus healthy individuals (HCs) as control groups. Quantification of extracted EVs was performed by nanoparticle tracking analysis and EVs RNA content was extracted using miRNeasy^®^ micro kit. At the initiation of the study, higher EV levels were observed in BCa versus HCs which although significantly increased during the treatment, however, reduced EVs levels down to HCs’ levels were observed 6 months after surgery. Further analysis was performed using RNA sequencing to identify whether gene expression profiling can be used to predict treatment outcomes. Based on the response to chemotherapy, Sadovska et al. subdivided participants into two separate responder and non-responder groups. The results indicated 4 lncRNAs, 6 miRNAs and one snoRNA were significantly higher in the non-responder group as well as HCs. In their study, induced some EVs-ncRNAs were observed as a consequence of chemotherapy. In conclusion, Sadovska et al. demonstrated EVs cargoes, especially EVS-ncRNAs are useful diagnostic and prognostic biomarkers in breast cancer patients undergoing neoadjuvant chemotherapy.

Kang et al. introduced three serum EVs-ncRNAs including hsa_circ_0001492, hsa_circ_0001439, and hsa_circ_0000896 as useful diagnostic biomarkers for lung adenocarcinoma. In their study, the expression levels of the mentioned ncRNAs in serum and serum EVs were measured using quantitative real-time PCR. Higher expression levels of EVs-ncRNAs in serum samples of patients with lung adenocarcinoma were associated with the progression of malignancy.

Taken together we hope this Research Topic about EV-ncRNAs comprises informative evidence for better understanding of the EV-ncRNAs and their importance in cancer biology.

## Author contributions

NN and AA-e-A were equally involved in drafting the work. FY, SA, and HP critically revised it. In addition, HP was the correspondent. All authors contributed to the article and approved the submitted version.
